# Association between serum estradiol levels and cognitive function in older women: a cross-sectional analysis

**DOI:** 10.3389/fnagi.2024.1356791

**Published:** 2024-02-21

**Authors:** Qian Xu, Meng Ji, Shicai Huang, Weifeng Guo

**Affiliations:** ^1^Suzhou Wujiang District Hospital of Traditional Chinese Medicine (Suzhou Wujiang District Second People's Hospital), Suzhou, China; ^2^Kunshan Integrated TCM and Western Medicine Hospital, Kunshan, China; ^3^First Clinical Medical College, Nanjing University of Chinese Medicine, Nanjing, China

**Keywords:** biomarker, estradiol, cognitive function, gerontology, national survey

## Abstract

**Introduction:**

Estradiol is a sex steroid hormone, which has been implicated in the pathogenesis of Alzheimer’s disease and cognitive impairment. This cross-sectional study aimed to examine the relationship between serum estradiol levels and cognitive performance in older American women.

**Methods:**

Data were obtained from the National Health and Nutrition Examination Survey 2013–2014. A total of 731 women aged ≥60 years who met the inclusion criteria were included in this study. Serum estradiol levels were measured using the isotope dilution liquid chromatography tandem mass spectrometry (ID-LC–MS/MS) method developed by the Centers for Disease Control and Prevention for routine analysis. All measured serum levels were further divided into three parts: T1, <3.68 pg./mL; T2, 3.68–7.49 pg./mL; T3, >7.49 pg./mL, and analyzed. Participants’ cognitive abilities were tested using the Vocabulary Learning Subtest (CERAD), Animal Fluency Test (AFS), and digital symbol substitution test (DSST). Scores for each test were calculated based on the sample mean and standard deviation (SD). To examine the relationship between serum estradiol level tertiles and cognitive scores, multiple linear regression models were developed, controlling for race/ethnicity, education level, hypertension, diabetes, and insomnia.

**Results:**

The mean age of the participants was 69.57 ± 6.68 years. The non-Hispanic whites were 78.95%, and those who had completed at least some college-level education were 60.62%. The mean BMI of the participants was 29.30 ± 6.79, and 10.85% had a history of smoking. Further, 73.41% did not have a history of alcohol consumption, and 63.03% had hypertension (63.03%). In addition, 81.81 and 88.3% did not have a history of diabetes mellitus and did not have sleep disorders, respectively. The mean serum estradiol level was 8.48 ± 0.77 pg./mL. Multivariate linear regression of the reference group consisting of participants in tertiles of serum estradiol levels revealed that one unit increase in serum estradiol levels increased DSST scores by 0.61 (0.87, 6.34) in the T3 group. However, no significant correlation was found in the CERAD and AFS tests.

**Conclusion:**

Participants with higher estradiol levels had higher DSST scores and better processing speed, sustained attention, and working memory, suggesting that serum estradiol may serve as a biomarker for cognitive decline in older women.

## 1 Introduction

Cognitive impairment is the most common neurodegenerative alteration in older adults (Islam, 2017). Its prominent clinical manifestation is a decline in memory, attention, language, and visuospatial abilities. Currently, >50 million people worldwide suffer from cognitive impairment, the majority of whom are older adults (Feigin et al., 2019). Age-related cognitive decline can serve as an early indicator of dementia, a condition that affects 5.1 million individuals in the United States. Notably, the prevalence of dementia is projected to increase two-fold by 2050 (Hebert et al., 2013). Further, the worldwide economic burden of dementia is expected to increase to USD 2.54 trillion by 2030 and approximately USD 1 trillion by 2050, which was USD 957.56 billion in 2015 (Jia et al., 2018). Cognitive impairment is linked to hereditary factors, dietary issues, mitochondrial malfunction, oxidative stress, and aging (Tobore, 2019). Cognitive impairment significantly diminishes the overall quality of life and imposes a substantial burden on society. Consequently, investigating the underlying variables that contribute to dementia is crucial to developing effective strategies for its prevention and treatment.

Estradiol, an estrogen synthesized by the ovaries and adrenal glands, plays a crucial role in the development of female sexual characteristics and fertility. Additionally, it can penetrate the blood–brain barrier and influence the brain (Brann et al., 2022). Estrogen is a steroid hormone and exists in three forms: estrone (E1), estradiol (E2), and estriol (E3). Among these, estradiol is the most prevalent in the human body. Estradiol plays a crucial role in controlling various physiological and pathological processes, including reproduction, sexual development, cancer pathogenesis, cognitive function, and neuroprotection (Azcoitia et al., 2018; Brocca and Garcia-Segura, 2019; Sahab-Negah et al., 2020; Brann et al., 2022). Although fluctuations in E2 levels during the normal menstrual cycle have no effect on women’s overall cognitive performance, the types of cognitive performance that women specialize in vary at different times during the menstrual cycle. During the pre-follicular phase (low estradiol levels), women have relatively enhanced spatial abilities, whereas women in the late follicular or mid-luteal phases (high estradiol levels) have relatively strong verbal fluency and memory abilities (Sundström Poromaa and Gingnell, 2014). Estradiol levels decline substantially during menopause and continue to decline annually with age (Rannevik et al., 1995). Decreased estradiol levels in older women affect memory, with significant reductions in both word recall and fluency (Weber et al., 2014), as well as in the ability to attend to objects and working memory (Schaafsma et al., 2010; Weber et al., 2014). As estrogen levels decline dramatically after menopause, women are more likely to develop Alzheimer’s disease and experience cognitive decline (Ryan et al., 2014; Pike, 2017; Boyle et al., 2021). Estrogen supplementation improves dementia symptoms (Wu et al., 2020). Nevertheless, some studies have demonstrated inconclusive findings, as changes in estrogen levels do not exhibit a significant correlation with immediate memory, delayed recall, language acquisition, or verbal fluency (Mihalj et al., 2014). Owing to the conflicting findings, we conducted an in-depth investigation into the precise impact of estradiol on cognitive performance in older women. We hypothesized that higher estradiol levels would be beneficial for cognitive functioning.

We examined a sample of older women, specifically those aged ≥60 years, representative of the entire nation. This study was conducted as part of the National Health and Nutrition Examination Survey (NHANES) to explore the correlation between serum estradiol levels and cognitive function.

## 2 Materials and methods

### 2.1 Data collection and study population

We retrieved publicly available data on female participants aged ≥60 years from a survey cycle of the 2013–2014 NHANES, conducted by the Centers for Disease Control and Prevention (CDC) and the National Center for Health Statistics (NCHS), to monitor the health status of the population in the United States. The NHANES protocol was approved by the NCHS Research Ethics Review Board, and all participants provided written informed consent. A cutoff age of ≥60 years was set based on the availability of serum estradiol concentration data. The exclusion criteria were male participants, participants aged <60 years, and those with missing estradiol data.

**Figure 1:**
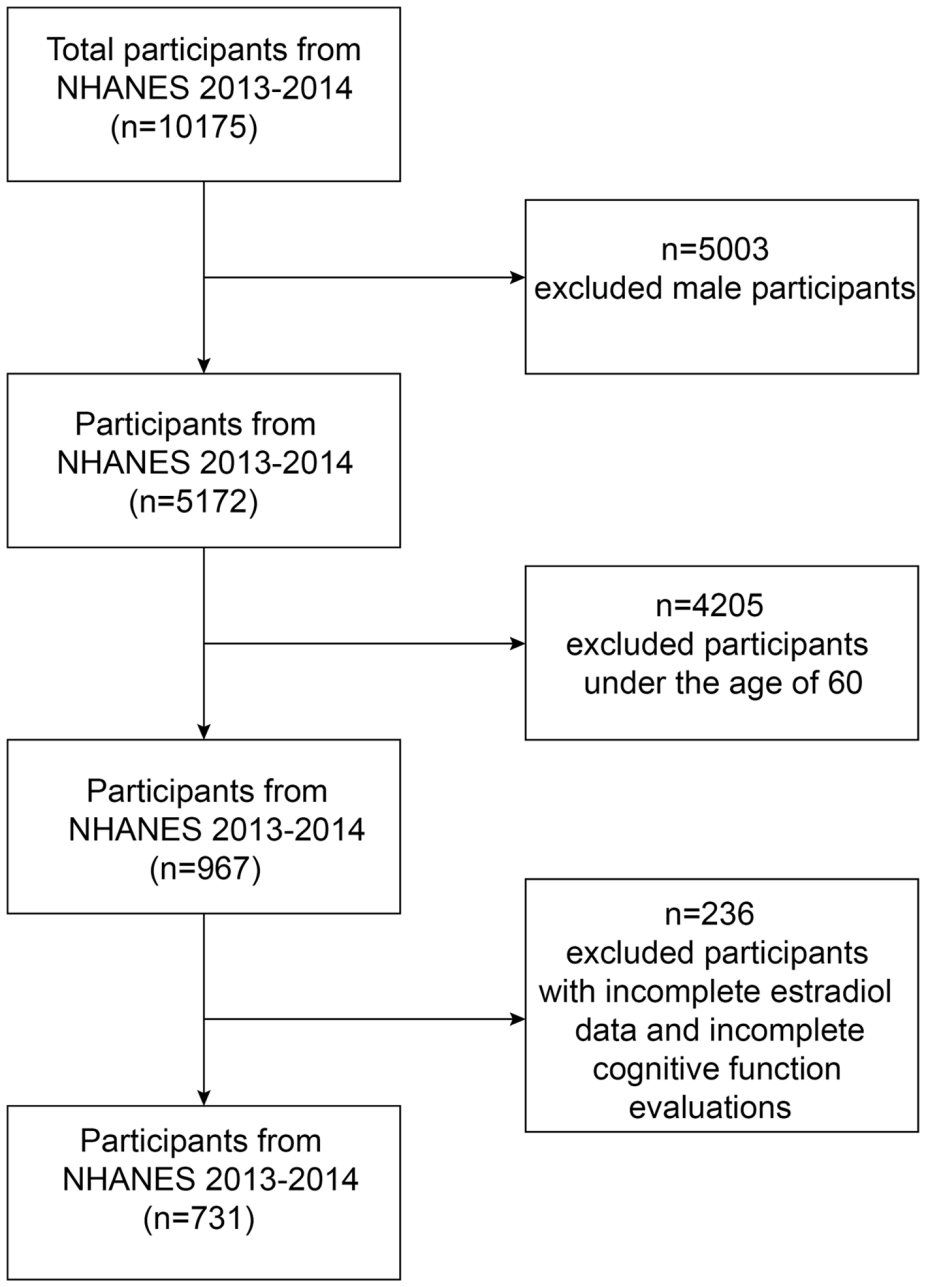
Flow chart of the screening process for the selection of eligible participants.

A total of 10,175 individuals were included in the study, and we limited our analysis to 967 females aged ≥60 years. Further, we excluded participants with true estradiol levels and incomplete cognitive assessment data (*n* = 236). Finally, 731 females were included in the study (Figure 1).

### 2.2 Cognitive performance assessment

The 2013–2014 NHANES Cognitive Function Data File contains three specific tests: the Alzheimer’s Disease Registry Association Vocabulary Learning Subtest (CERAD), Animal Fluency Test (AFS), and Digit Symbol Substitution Test (DSST). Although cognitive tests cannot replace diagnoses made through clinical examinations, they are employed in large-scale screening and epidemiological investigations (Fillenbaum et al., 2008; Gao et al., 2009).

The CERAD test assesses immediate and delayed learning of linguistic information (memory subdomains) (Morris et al., 1994), with word recall delays following the completion of the other two cognitive exercises (AFS and DSST) (approximately 8–10 min from the beginning of the word learning trials). Scores after recall delays were used as the results of the CERAD test in this study.

AFS assesses explicit language category fluency, which is a constituent of executive function (Strauss et al., 2006), along with other functions, including semantic memory and processing speed (Clark et al., 2009). Participants were asked to name as many animals as possible in 1 min and assign each animal a score.

The DSST is a component of the Wechsler Adult Intelligence Scale (WAIS III), which assesses processing speed, sustained attention, and working memory (Wechsler, 1997). Higher scores on each examination indicate a higher level of cognitive functionality in individuals. This examination was conducted using a physical document comprising a set of nine numbers accompanied by distinct symbols positioned at the uppermost section of the form. Participants were asked to replicate the relevant symbols found in the 133 boxes located next to the numbers in 2 min.

For more information on scoring, see the 1999–2000 NHANES CFQ Questionnaire Data File document at https://wwwn.cdc.gov/Nchs/Nhanes/1999-2000/CFQ.htm.

### 2.3 Serum estradiol assessment

Every participant underwent venipuncture, which involved drawing blood in the morning after an overnight fast. Serum samples were processed, stored, and sent to the National Center for Environmental Health, CDC, Division of Laboratory Sciences in Atlanta, Georgia, for examination. An isotope dilution liquid chromatography tandem mass spectrometry (ID-LC–MS/MS) method developed by the CDC was used to perform routine serum estradiol assays. Thereafter, the serum levels were divided into three equal halves: T1, estradiol level < 3.68 pg./mL; T2, estradiol level 3.68–7.49 pg./mL; T3: estradiol level > 7.49 pg./mL, and evaluated.

### 2.4 Covariates

Data on covariates pertaining to estradiol and cognitive performance were collected, encompassing demographic factors including age, race, and education level. The questionnaire results provided a comprehensive range of demographic information that was subsequently transformed into relevant categorical variables. Smoking, alcohol consumption, BMI, diabetes, hypertension, and sleep disorders were identified as potential confounding factors.

### 2.5 Statistical analysis

The surveys relied on accurate weights for intricate surveys directly supplied by the NHANES. Continuous variables were presented as the mean ± standard deviation (SD), whereas categorical variables were presented as totals and percentages (%). The chi-square test was used to analyze categorical variables. Initially, the normality of continuous variables was assessed. A one-way analysis of variance (ANOVA) was performed for normally distributed data, whereas the Kruskal–Wallis test (a nonparametric ANOVA test) was performed for a non-normal distribution. This study evaluated the relationship between estradiol levels and cognitive function using both unadjusted and multifactor-adjusted models through generalized logistic regression. To investigate and comprehend the intricate correlation between estradiol and cognitive function, the continuous variables were transformed into categorical variables: estradiol levels <3.68 pg./mL, 3.68–7.49 pg./mL, and > 7.49 pg./mL. Multivariate models were then adjusted to account for age, ethnicity, education, BMI, smoking status, alcohol consumption, and comorbidities such as hypertension and diabetes. Statistical significance was set at *p* < 0.05. All statistical analyses were performed using the R statistical package (version 3.5.3) and EmpowerStats.

## 3 Results

### 3.1 Characteristics of the study population based on serum estradiol levels

The study comprised 731 females aged ≥60 years, with a mean age of 69.57 ± 6.68. We observed a negative correlation between age and estradiol levels, indicating that estradiol levels decrease with age. This information is presented in Table 1. Non-Hispanic whites comprised the highest proportion of participants with varying estradiol levels. Further, serum estrogen levels exhibited an inverse correlation with BMI. A strong positive correlation was observed between blood estradiol levels and the number of patients with diabetes. Nevertheless, no notable differences were observed for education level, smoking, alcohol consumption, hypertension, or sleep disorders. In the population baseline table, no significant difference was observed (*p* > 0.05) between the CERAD and AFS tests and serum estradiol concentrations. However, a significant difference was observed between DSST scores and serum estradiol concentrations. The higher the estradiol concentration, the higher the DSST score (*p* < 0.05).

**Table 1:** Baseline characteristics of participants.

	Total *N* = 731	Estradiol tertiles (pg/mL)			*p-*value
		T1 (<3.68) *N* = 243	T2 (≥3.68to ≤ 7.49) *N* = 244	T3 (>7.49) *N* = 243	
Age (y), Mean (SD)	69.57 (6.68)	71.10 (6.65)	68.81 (6.35)	68.93 (6.79)	0.0001
Race, *n* (%)					0.0024
Mexican American	31 (4.11%)	14 (5.7%)	9 (3.56%)	8 (3.17%)	
Other Hispanic	24 (3.42%)	9 (3.84%)	8 (3.41%)	7 (3.03%)	
Non-Hispanic White	577 (78.95%)	186 (76.55%)	200 (82.12%)	190 (77.89%)	
Non-Hispanic Black	65 (8.83%)	13 (5.37%)	18 (7.39%)	33 (13.6%)	
Other Race	34 (4.7%)	21 (8.54%)	9 (3.52%)	6 (2.31%)	
Education, *n* (%)					0.7361
Below high school	117 (16.09%)	43 (17.8%)	38 (15.7%)	36 (14.87%)	
High school graduate	171 (23.29%)	59 (24.18%)	60 (24.4%)	62 (21.29%)	
College graduate or above	443 (60.62%)	141 (58.01%)	146 (59.9%)	156 (63.83%)	
BMI (kg/m^2^), Mean (SD)	29.30 (6.79)	25.44 (4.42)	28.82 (5.45)	33.42 (7.55)	<0.0001
Smoking, *n* (%)					0.0828
Yes	79 (10.85%)	36 (14.69%)	27 (10.98%)	17 (7.08%)	
No	223 (30.46%)	67 (27.49%)	71 (29.56%)	84 (34.2%)	
Missing	429 (58.69%)	140 (57.82%)	145 (59.46%)	143 (58.71%)	
Drinking, *n* (%)					0.678
Yes	47 (6.47%)	15 (5.95%)	15 (7.12%)	15 (6.26%)	
No	537 (73.41%)	177 (72.93%)	177 (75.66%)	175 (71.51%)	
Missing	147 (20.12%)	51 (21.12%)	51 (17.21%)	54 (22.23%)	
Hypertension, *n* (%)					0.0553
Yes	461 (63.03%)	144 (59.26%)	148 (60.68%)	168 (69.03%)	
No	270 (36.97%)	99 (40.74%)	76 (39.32%)	76 (30.97%)	
DM, *n* (%)					0.0313
Yes	133 (18.19%)	43 (17.9%)	56 (22.76%)	33 (13.69%)	
No	598 (81.81%)	200 (82.1%)	188 (77.24%)	211 (86.31%)	
Sleeping disorder, *n* (%)					0.5429
Yes	86 (11.7%)	26 (10.8%)	26 (10.73%)	33 (13.55%)	
No	645 (88.3%)	217 (89.2%)	218 (89.27%)	211 (86.45%)	
CERAD(score)	6.79 (2.37)	6.67 (2.53)	6.80 (2.23)	6.90 (2.34)	0.5675
Animal fluency test(score)	17.78 (5.35)	17.37 (5.58)	17.64 (4.98)	18.32 (5.47)	0.1353
DSST(score)	53.48 (16.96)	50.40 (15.57)	54.29 (16.66)	55.55 (18.07)	0.0026

Mean (SD) for: Age BMI CERAD Animal fluency test DSST. *p* value was calculated by weighted linear regression model.(%) for: Race Education Smoking Drinking Hypertension DM. *p* value was calculated by weighted chi-square test. BMI, body mass index; DM, diabetes mellitus; CERAD, Consortium to Establish a Registry for Alzheimer’s disease; DSST, Digit Symbol Substitution test; T, tertile.

### 3.2 Correlation between serum estradiol levels and cognitive function

Table 2 shows the outcomes of the multivariate regression analysis of serum estradiol levels and cognitive performance. We employed three multivariate logistic regression models to demonstrate the correlation between serum estradiol levels and cognitive performance. Model 1 is the unadjusted model; that is, it does not consider any covariates. Model 2 is adjusted for age and ethnicity. Model 3 is adjusted for age, ethnicity, educational level, BMI, hypertension, diabetes mellitus, alcohol consumption, cigarette smoking, and sleep disorders. We found no significant correlation between estradiol levels and cognitive function tests for the CERAD and AFS in older women. However, a substantial positive association was observed between estradiol levels and DSST scores. After controlling for several factors, such as age, race, sex, education level, hypertension, body mass index, smoking, stroke, alcohol consumption, and diabetes, the observed correlation remained stable across all multivariate logistic regression models. In the DSST, a significant increase was observed in the DSST score of 0.61 (0.87, 6.34), *p* = 0.0099, for every incremental unit of serum estradiol (pg/mL) in the T3 group compared with the T1 group, after adjusting for confounding factors.

**Table 2:** Associations between tertile of estradiol (reference: <3.68 pg./mL) and CREAD/DSST/AFS test score, β (95%CI) *p*-value.

Estradiol (pg/mL)	Tertile of estradiol			*p* for trend
	T1 (<3.68)	T2 (≥3.68to ≤ 7.49)	T3 (>7.49)	
CERAD test				
Crude	1.00 (Ref.)	0.13 (−0.29, 0.56) 0.5309	0.23 (−0.20, 0.66) 0.2889	0.02 (−0.02, 0.07) 0.3048
Model 1	1.00 (Ref.)	−0.10 (−0.51, 0.32) 0.6464	0.05 (−0.37, 0.47) 0.8185	0.01 (−0.04, 0.05) 0.7144
Model 2	1.00 (Ref.)	−0.06 (−0.48, 0.36) 0.7885	0.02 (−0.45, 0.49) 0.9364	0.00 (−0.05, 0.06) 0.8836
DSST test				
Crude	1.00 (Ref.)	3.89 (0.89, 6.89) 0.0113	5.15 (2.11, 8.18) 0.0009	0.52 (0.19, 0.85) 0.0021
Model 1	1.00 (Ref.)	0.82 (−1.76, 3.39) 0.5344	3.06 (0.44, 5.68) 0.0224	0.35 (0.06, 0.63) 0.0172
Model 2	1.00 (Ref.)	1.12 (−1.30, 3.55) 0.3648	0.61 (0.87, 6.34) 0.0099	0.40 (0.11, 0.70) 0.0079
AFS test				
Crude	1.00 (Ref.)	0.27 (−0.68, 1.22) 0.5751	0.95 (−0.01, 1.91) 0.0535	0.11 (0.00, 0.21) 0.0460
Model 1	1.00 (Ref.)	−0.39 (−1.29, 0.52) 0.4028	0.52 (−0.39, 1.44) 0.2636	0.07 (−0.03, 0.17) 0.1495
Model 2	1.00 (Ref.)	−0.15 (−1.03, 0.74) 0.7454	0.80 (−0.19, 1.80) 0.1142	0.10 (−0.01, 0.21) 0.0702

Model 1 adjusted for age, race. Model 2 adjusted for age, race, education, BMI, smoking, drinking, DM, sleeping disorder.

### 3.3 Stratified relationship between serum estradiol levels and cognitive function

Table 1 shows that BMI and diabetes were significantly associated with serum estradiol levels. We further analyzed the hierarchical relationship between serum estradiol levels and cognitive function for different BMI levels and diabetes history subgroups (Tables 3, 4). Hierarchical analysis showed that participants with no history of diabetes scored higher in the cognitive function tests (CERAD, AFS, and DSST) than those with diabetes when one unit of serum estradiol was added. We divided the participants into non-overweight and overweight groups based on their BMI. Compared with overweight participants, non-overweight participants had higher DSST scores for every one-unit increase in serum estradiol levels, but this was not observed in the CERAD and AFS tests. Further, no statistically significant differences were observed in the interaction tests for these hierarchical relationships (*p* > 0.05).

**Table 3:** Stratified association between serum estradiol and cognitive function testing.

Serum estradiol (pg/mL)	*N*	β (95% CI) *p*-value	*P*-interaction
For CEARD			
Diabetes Yes	133	0.01 (−0.08, 0.11) 0.7909	0.8556
Diabetes No	598	0.00 (−0.00, 0.01) 0.1123	
For AFS			
Diabetes Yes	133	0.08 (−0.12, 0.27) 0.4489	0.4925
Diabetes No	598	0.01 (−0.00, 0.02) 0.1153	
For DSST			
Diabetes Yes	133	0.20 (−0.34, 0.75) 0.4654	0.4915
Diabetes No	598	0.02 (−0.01, 0.05) 0.2925	

Above adjusts for age, race, education, BMI, smoking, drinking, sleeping disorder. In each case, the model was not adjusted for the stratification variable itself.

**Table 4:** Stratified association between serum estradiol and cognitive function testing.

Serum estradiol (pg/mL)	*N*	β(95% CI) *p*-value	*P* interaction
For CEARD			
Underweight to normal weight	158	0.00 (−0.01, 0.02) 0.3680	0.8228
Overweight to obesity	573	0.00 (−0.00, 0.01) 0.2333	
For AFS			
Underweight to normal weight	158	0.00 (−0.02, 0.03) 0.6772	0.7461
Overweight to obesity	573	0.01 (−0.00, 0.02) 0.1552	
For DSST			
Underweight to normal weight	158	0.04 (−0.02, 0.11) 0.1546	0.2343
Overweight to obesity	573	0.00 (−0.03, 0.04) 0.8551	

Above adjusts for age, race, education, DM, smoking, drinking, sleeping disorder. In each case, the model was not adjusted for the stratification variable itself.

## 4 Discussion

Our findings reveal that participants with elevated estradiol levels demonstrated superior performance in the DSST, as well as enhanced processing speed, sustained attention, and working memory. However, no notable differences were observed in the AFS and CERAD test results. These findings indicate that serum estradiol levels may serve as a biomarker of cognitive decline in older individuals.

As estradiol affects women’s cognitive function at different stages of the menstrual cycle, higher estradiol levels can enhance memory in women (Sundström Poromaa and Gingnell, 2014). Notably, the DSST mainly involves replicating the symbols adjacent to numbers, thereby assessing the memory of an individual. Nevertheless, no notable differences in the AFS and CERAD tests may be attributed to the small sample size, and larger sample surveys in the future may have different outcomes.

The precise molecular mechanisms underlying the association between estradiol levels and cognitive function are currently being studied. Estradiol enhances neurotransmitter metabolism, production of neurotrophins, and formation of synapses in the hippocampus and prefrontal cortex. These brain regions are closely associated with memory consolidation and enhanced cognitive function (Luine, 2014). Cognitive dysfunction may occur due to reduced acetylcholine and its receptor levels, whereas estrogen enhances acetylcholine synthesis and mitigates cholinergic neuronal damage (Rabbani et al., 1997). Estrogens exert neuroprotective effects by mitigating oxidative stress, scavenging oxygen-free radicals, and diminishing and postponing neuronal aging (Chakrabarti et al., 2016). miR-125b, a highly prevalent miRNA in the brain, provides protection against cortical neuronal toxicity caused by Aβ (β-amyloid). Additionally, estradiol enhances miR-125b expression, which in turn inhibits genes that promote cell death, thereby reducing brain neuronal toxicity (Micheli et al., 2016; Amakiri et al., 2019). Notably, estradiol plays a crucial role in controlling the growth and survival of cells in the hippocampus, which is responsible for memory and learning (Duarte-Guterman et al., 2015). The hippocampus is more sensitive to changes in estradiol levels than other brain regions (Pletzer et al., 2018). Fluctuations in E2 levels during the female sexual cycle cause dynamic changes in hippocampal volume and dendritic spine density (Protopopescu et al., 2008; Pletzer et al., 2018). However, whether these changes are beneficial for DSST remains unclear and warrants further investigation.

In addition to estrogen, other sex hormones can also affect cognitive function. A population-based study conducted on androgens suggested that older men with higher testosterone levels demonstrated better performance in multiple cognitive function tests (Hsu et al., 2015). Further, no significant association was observed between changes in androgen levels and cognitive function in a 23-year-long prospective cohort study of 3,044 women (Koyama et al., 2016), and no significant correlation was observed between total testosterone levels and cognitive performance in later life. Longitudinal data from 4,110 study participants (Kische et al., 2017) showed that serum testosterone levels were not associated with cognitive function in older women. Overall, testosterone levels in women are much lower than those in men, and even during menopause, fluctuations in testosterone levels are relatively small, which may explain the above findings. Notably, hormone levels greatly fluctuate in perimenopausal women, and memory problems are also one of the most common complaints among menopausal women. Early supplementation of progesterone after menopause can improve speech memory and other functions in women (Alhola et al., 2010); however, the impact of progesterone on cognitive function in women >60 years of age remains unclear.

This study has several strengths. The study population consisted of older individuals who are representative of the entire nation, thereby increasing the applicability of our results. Furthermore, this research targeted a specific population—older women—with heightened susceptibility to cognitive decline. Nevertheless, this study also has some limitations. First, the cross-sectional design of this study is the primary limitation. Consequently, we could not establish a cause-and-effect relationship or observe any variation in estradiol levels or cognitive performance in the older women throughout the study period. Second, confounding factors, such as depressive symptoms, which may have influenced our study results, were not included in the analyses. Notably, depressive symptoms were not documented in the 2013–2014 NHANES. Third, as these data were gathered during 2013–2014, they may no longer be relevant and may not accurately represent the current estradiol levels and cognitive performance in older individuals. Finally, the NHANES survey may lack a comprehensive representation of specific categories, including rural communities, homeless individuals, and non-native English speakers. Subsequent investigations must incorporate a longitudinal framework to scrutinize the correlation between estradiol levels and cognitive function in older women, particularly in non-Western nations.

In summary, our results revealed a significant correlation between estradiol levels and cognitive function in older women. Our study indicates that estradiol levels could potentially be used as a biomarker to measure cognitive function deterioration in older individuals.

## Data availability statement

The original contributions presented in the study are included in the article/supplementary material, further inquiries can be directed to the corresponding author.

## Ethics statement

The studies involving human participants were reviewed and approved by the NHANES has been approved by the National Center for Health Statistics Research Ethics Review Board. The patients/participants provided their written informed consent to participate in this study. Written informed consent was obtained from the individual(s) for the publication of any potentially identifiable images or data included in this article.

## Author contributions

QX: Formal analysis, Investigation, Writing – original draft, Writing – review & editing. MJ: Conceptualization, Investigation, Methodology, Software, Writing – original draft. SH: Data curation, Formal analysis, Validation, Writing – review & editing. WG: Data curation, Project administration, Supervision, Validation, Writing – review & editing.
